# On the Role of Contrast Polarity: In Response to van der Helm’s Comments

**DOI:** 10.3390/brainsci10010054

**Published:** 2020-01-17

**Authors:** Baingio Pinna, Livio Conti

**Affiliations:** 1 Department of Biomedical Science, University of Sassari, 07100 Sassari, Italy; 2 Faculty of Engineering, Uninettuno University, 00186 Roma, Italy; livio.conti@uninettunouniversity.net

**Keywords:** amodal completion, contrast polarity, simplicity principle, likelihood principle, Bayes’ framework

## Abstract

In this work, we discussed and counter-commented van der Helm’s comments on our previous paper (Pinna and Conti, *Brain Sci.*, **2019**, *9*, 149), where we demonstrated unique and relevant visual properties imparted by contrast polarity in eliciting amodal completion. The main question we addressed was: “What is the role of shape formation and perceptual organization in inducing amodal completion?” To answer this question, novel stimuli were studied through Gestalt experimental phenomenology. The results demonstrated the domination of the contrast polarity against good continuation, T-junctions, and regularity. Moreover, the limiting conditions explored revealed a new kind of junction next to the T- and Y-junctions, respectively responsible for amodal completion and tessellation. We called them I-junctions. The results were theoretically discussed in relation to the previous approaches and in the light of the phenomenal salience imparted by contrast polarity. In counter-commenting van der Helm’s comments we went into detail of his critiques and rejected all of them point-by-point. We proceeded by summarizing hypotheses and discussion of the previous work, then commenting on each critique through old and new phenomena and clarifying the meaning of our previous conclusions.

## 1. Introduction

Van der Helm [1] commented in a surprisingly polemical way on a recent paper of ours [2]. In this work, we answer his comments by proceeding in an objective manner on the basis of what was truly written and demonstrated in that paper on the basis of mere phenomenal evidence. In addition to the previous conditions, several others will be shown to make even more clear to the reader our previous purposes and conclusions, as well as the following counter-comments.

Our reply will be organized into sections based on van der Helm’s comments. However, primarily for the sake of clarity, a short summary of Pinna and Conti [2] is required. This is useful to correctly inform readers about our previous purposes and conclusions necessary for a better understanding of our responses.

## 2. Pinna and Conti (2019) in Short

In this work, we demonstrated unique and relevant visual properties imparted by contrast polarity in perceptual organization and, more particularly, in eliciting amodal completion, where T-junction, good continuation, and closure are considered as the main factors involved.

Some of the most relevant explanations of amodal completion based on Helmholtz’s likelihood principle [3] and Gregory’s “unconscious inference” [4] were discussed. In short, the amodal object is similar to a perceptual hypothesis postulated to explain the unlikely gaps within the pattern of stimuli and the one that most likely produces the sensory stimulation (cf. the so-called avoidance-of-coincidences principle [5,6]). More recently these approaches have been reconsidered in terms of probabilistic Bayesian inference, applied successfully in many classical amodal conditions.

Another approach discussed within the paper is the simplicity–Prägnanz principle of Gestalt psychologists, according to which the visual system is aimed at finding the simplest and most stable organization consistent with the sensory inputs.

These approaches focus on the shape completed behind the occluder by assuming amodal completion as the cause of the formation of the shape partially occluded. Therefore, the main interest is to account for how the occluded object is completed, what is the amodal shape, and how contours of partially visible fragments are relatable behind an occluder.

Differently, we adopted a complementary perspective, assuming amodal completion not as the cause but as the resulting effect. Therefore, the questions are now the following: What is the role of shape formation and perceptual organization in inducing amodal completion? What are the perceptual conditions that elicit the segregation of occluded and occluding objects and, finally, amodal completion? What is the role of the local contours, junctions, and termination attributes in eliciting the phenomenon of amodal completion?

Within this perspective, amodal completion was considered as a visual phenomenon not as a process, i.e., the final outcome of perceptual processes and grouping principles. Therefore, the contrast polarity has been used as the main grouping and ungrouping factor aimed to explore and test amodal completion as a visual phenomenon elicited by good continuation, T-junctions, closure, and regularity.

Together with traditional configurations, novel stimuli, which have been reduced more and more to extreme limiting conditions, were introduced.

Phenomenally, we showed that contrast polarity is effective in inducing amodal completion in conditions where, on the basis of the known principles and of the previous theoretical approaches, it is not expected and vice versa. In this way, amodal completion was annulled or disrupted in the stimuli where it was supposed to be effective. More particularly, contrast polarity has been able to elicit/disrupt amodal completion when pitted against or in favor of the following conditions reduced to limiting cases: (i) Classical patterns (Figures 7 and 8); (ii) Petter’s effect and Petter’s rule (Figures 9–11); (iii) tessellation with T-junctions replaced by Y-junctions (Figures 12–16); (iv) group of isolated figures arranged in a cross (Figures 17–19); and (v) a single shape (Figures 20–22).

Our results demonstrated the compelling domination of the contrast polarity against good continuation, T-junctions, closure, and regularity. Moreover, the limiting conditions explored revealed a new kind of junction next to the T- and Y-junctions, respectively responsible for amodal completion and tessellation. They were called I-junctions, since eliciting amodal continuation of contours behind a contour with the same orientation.

Under our conditions, contrast polarity was shown to operate locally, eliciting results that could be independent from global scale and also paradoxical. We suggested that these results weaken and challenge theoretical approaches based on notions like oneness, unitariness, symmetry, regularity, simplicity, likelihood, constraints, and past knowledge. Moreover, Helmholtz’s likelihood principle, simplicity/Prägnanz, and Bayes’ inference are clearly questioned since they are supposed to operate especially at a global and holistic level of vision.

We proposed an alternative explanation of the specific outcomes based on the phenomenal dynamics made explicit by contrast polarity. First of all, the contrast polarity was perceived like a barrier analogous to, but stronger than, the one created by T-junctions. In other terms, the phenomenal salience, elicited by the highest luminance contrast going from black to white on a gray background, triggers a process of object segregation and unilateral belongingness of the boundaries.

The dominance of contrast polarity over good continuation, closure, regularity, and T-junctions is related to its phenomenal salience and highlighting effect. The same argument can account for the emergence of amodal continuation on I-junction, groups of isolated figures, and single shapes. Moreover, the imparted salience can disrupt, both locally and globally, arrangements of figures or can alternately rearrange the elements according to their similarity/dissimilarity. The highlighting strength of contrast polarity determines even the grouping effectiveness against the global and holistic rules and factors.

In favor of the basic and essential role of the phenomenal salience, we invoked deceiving strategies used in nature by most living organisms. Flowers, birds, and fishes use colors and contrast polarity to attract, reject, show, and hide. We suggest that the phenomenal salience strongly improves the biological fitness of living organisms and, therefore, the capability of an individual of a certain genotype to reproduce and, thus, to propagate an individual’s genes within the genes of the next generation.

The phenomenal salience is a basic requirement also in human beings, in the way we dress, invent fashion and design, in the way we use the maquillage, and in the existence of the maquillage itself.

The strength of phenomenal salience imparted by contrast polarity enables the full independence from local or global organizations and top-down or bottom-up dynamics. It eludes all these categories since it can play in favor or against each of them, as demonstrated in our stimuli. It represents a true challenge for the theories discussed here, which cannot easily incorporate it (e.g., as a prior) without losing explanatory power somewhere else. Inside these arguments, it is important to underline that phenomenal salience is a perceptual attribute not restricted to contrast polarity, but it can also be triggered by color, shape, motion, and every other visual property. Among them, contrast polarity is one of the most powerful.

Given the significance of this attribute, further experimental studies based on phenomenological, psychophysical, and neurophysiological techniques are required to measure the strength of the phenomenal salience imparted by contrast polarity against other attributes involved in amodal completion. Further studies can shed light on the role of contrast polarity as a general tool useful in testing the range of scientific effectiveness of visual theories, approaches, and models.

This summary is taken on purpose almost verbatim from the final Discussion section of Pinna and Conti.

To make clear these hypotheses and, above all, to demonstrate the partial and incorrect interpretations and judgments of van del Helm, it is necessary to show the way we operate with reversed contrast. This is a novel way that cannot be reduced to the examples reported by van der Helm. This is the topic of the next section.

## 3. Phenomenology of Contrast Polarity

**Figure 1:**
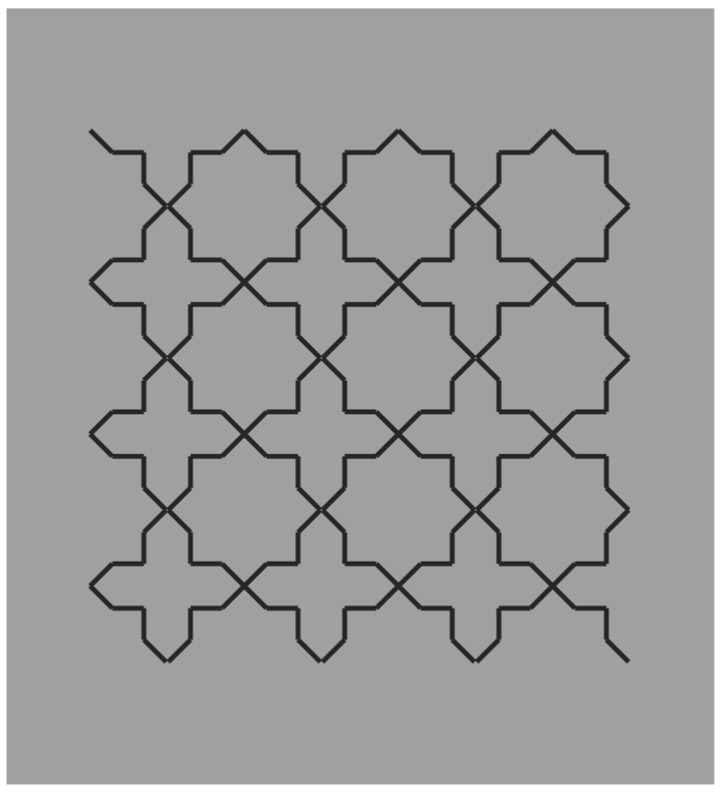
Crosses or stars? Alternated figure-ground segregation.

Van der Helm wrote: “The role of contrast polarity in human visual perception is a long-standing research topic (see, e.g., [7,8,9,10]). As a consequence, the phenomena presented by Pinna and Conti are not really as novel or surprising as they suggested them to be, but they are indeed illustrative of the effects of, in particular, contrast polarity reversals. For example, Figure 1 shows, in the style of Pinna and Conti, a stimulus in which such a reversal triggers a substantial change in the way in which it is perceptually organized.”

As mentioned in the previous section, contrast polarity has been used in our paper as a grouping/ungrouping factor aimed to explore and test amodal completion as a visual phenomenon elicited by good continuation, T-junctions, closure, and regularity. It is no coincidence that the title of our work is: “The Limiting Case of Amodal Completion: The Phenomenal Salience and the Role of Contrast Polarity.” A first difference in relation to the works [3,4,5,6,7], mentioned by van der Helms, is in the purposes but, more importantly, in the pattern of stimuli. In fact, while it is true that the quoted papers studied the role of contrast polarity, none of them used this factor the way we did, as can be easily demonstrated through their reading and especially by comparing their conditions with ours. 

This entails that, differently from van der Helm’s words—“The role of contrast polarity in human visual perception is a long-standing research topic (see, e.g., [7,8,9,10]). As a consequence, the phenomena presented by Pinna and Conti are not really as novel or surprising as they suggested them to be….”)—the conclusion, “As a consequence,” cannot be a correct logical implication. The fact that other scientists studied “the role of contrast polarity in human visual perception” does not imply that our conditions are not new. In fact, although the first proposition is true, the second is not necessarily true and false, as we are going to demonstrate. As a consequence, the resulting implication is false according to the associated logic truth table. 

This is not enough for a proper defense. We will now demonstrate the novelty of our conditions by showing some of them with further supporting examples. We will proceed going from known and trivial cases, as the one shown by van der Helm, to more and more interesting cases and demonstrations. 

We start again from van der Helm’s words already quoted: “…For example, Figure 1 shows, in the style of Pinna and Conti, a stimulus in which such a reversal triggers a substantial change in the way in which it is perceptually organized.” 

The example reported is only partially in our style. To be more precise, it is just the starting condition that in our paper became much more complex figure after figure. Van der Helm played here with perceptual grouping by pitting similarity against good continuation. This is a classical procedure used by Gestalt psychologists. A similar basic condition is illustrated in the following stimulus (cf. Figure 1).

Here, we do not perceive individual segments, unconnected and oriented in different directions, but we see two main alternated and complementary shapes: Crosses and eight-pointed stars. By perceiving the crosses, the stars are invisible and vice versa. This is related to the unilateral belongingness of the boundaries. The two main results of Figure 1 are reversible and they can be easily switched by changing the focus of attention or just by moving the gaze in different locations of the stimulus pattern. 

**Figure 2:**
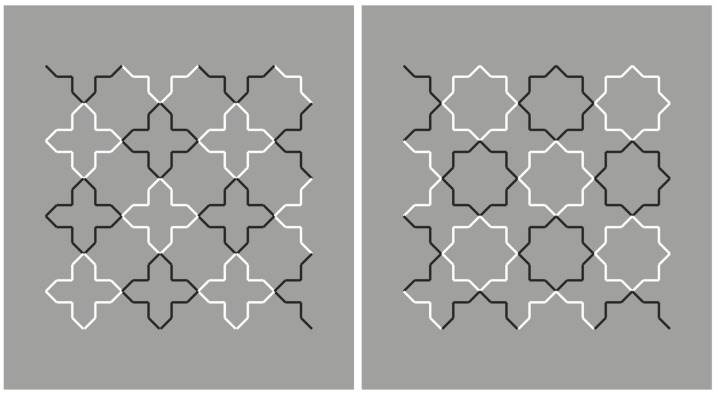
Crosses and stars due to the similarity principle of contrast polarity.

By introducing the grouping principle of similarity on the basis of the reversed luminance contrast, the salience of the crosses is now enhanced to the detriment of the stars (Figure 2, left). The opposite outcome is perceived in Figure 2, right. 

**Figure 3:**
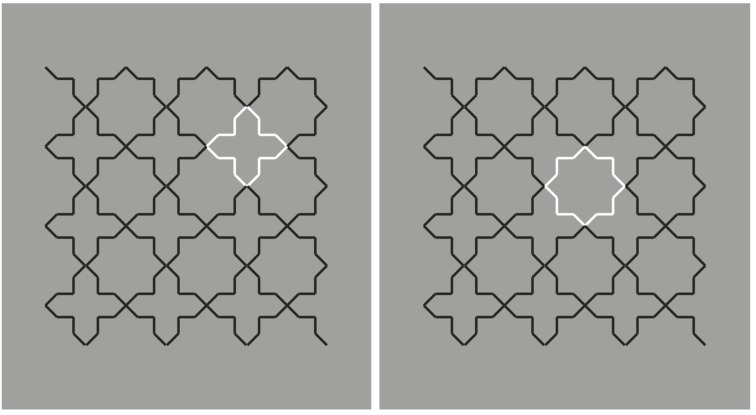
Crosses and stars spreading and filling in.

The story becomes more interesting in Figure 3, where, reversing the contrast of only one of the two objects is sufficient to highlight and, thus, elicit the emergence of all the other similar objects. The accentuation of one object (e.g., one cross) spreads to all the others (all the remaining crosses). This figure suggests that reversed contrast could act as an accentuation principle [11,12,13,14,15,16] and not just as similarity.

**Figure 4:**
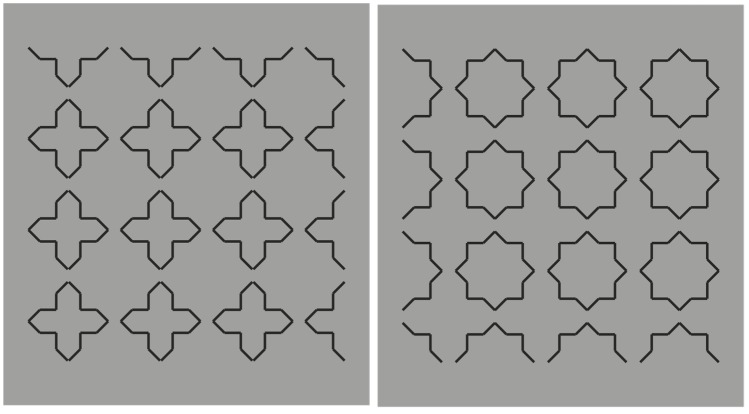
Crosses and stars elicited by the closure and proximity principles.

The strong connection between figure-ground segregation and grouping is clearly shown in Figure 4, where the closure and proximity principles induce a clear object segmentation: Stars on the left and crosses on the right.

**Figure 5:**
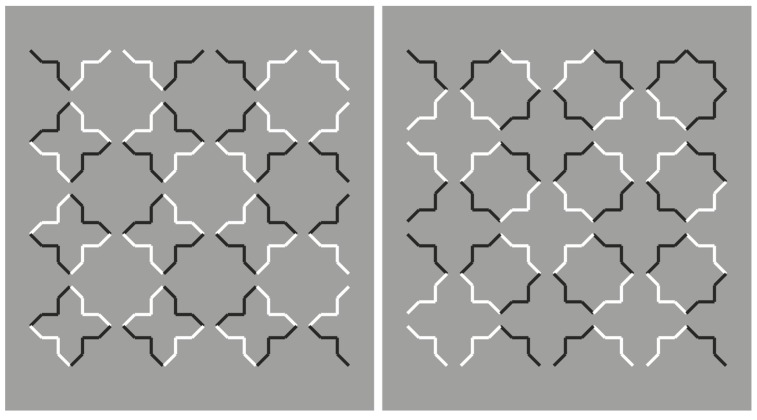
Similarity by contrast polarity pitted against closure and proximity principles.

In Figure 5, contrast polarity enables the emergence of the complementary regions or, at least, it weakens the results of Figure 4.

**Figure 6:**
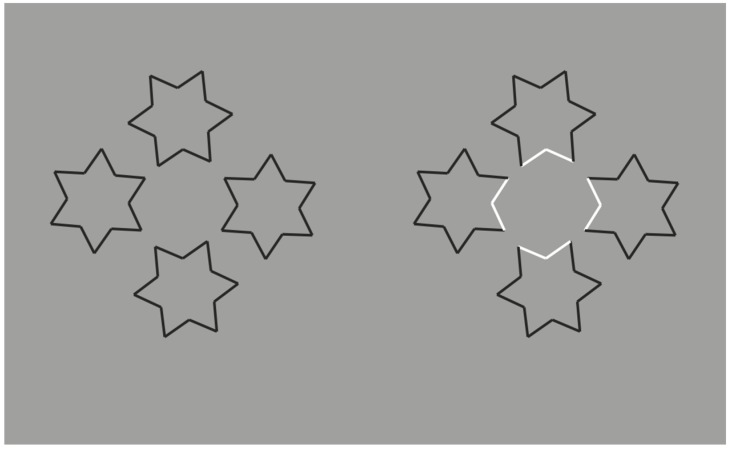
Similarity by contrast polarity pitted against closure and proximity principles.

Figure 6 shows a further and more interesting example, already reported within the paper.

**Figure 7:**
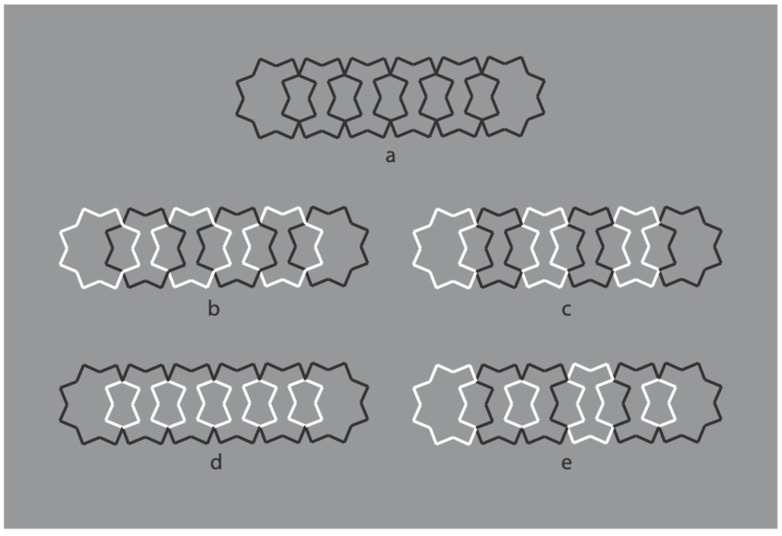
New complex results by overlapping six eight-pointed stars.

Further conditions are illustrated in Figure 7. Here, by overlapping six stars, the grouping of the components, recombined by contrast polarity, becomes even more complex and unexpected. 

Under these conditions, the regularity and homogeneity of the resulting object is impaired.

**Figure 8:**
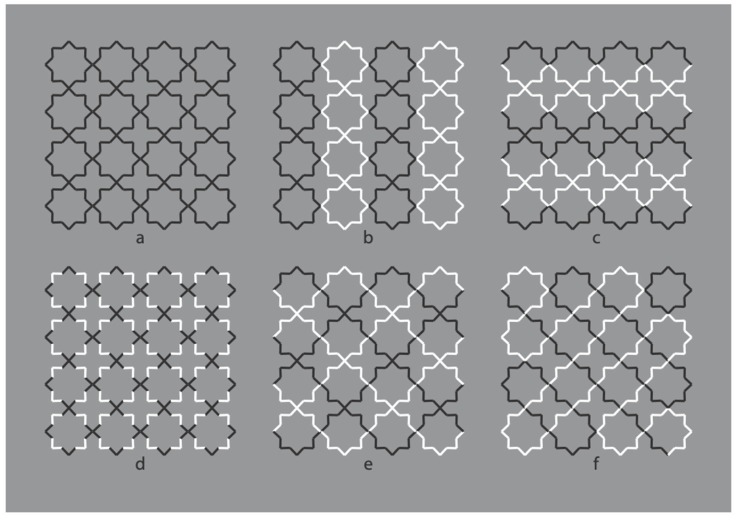
New regular and simpler results elicited by contrast polarity.

**Figure 9:**
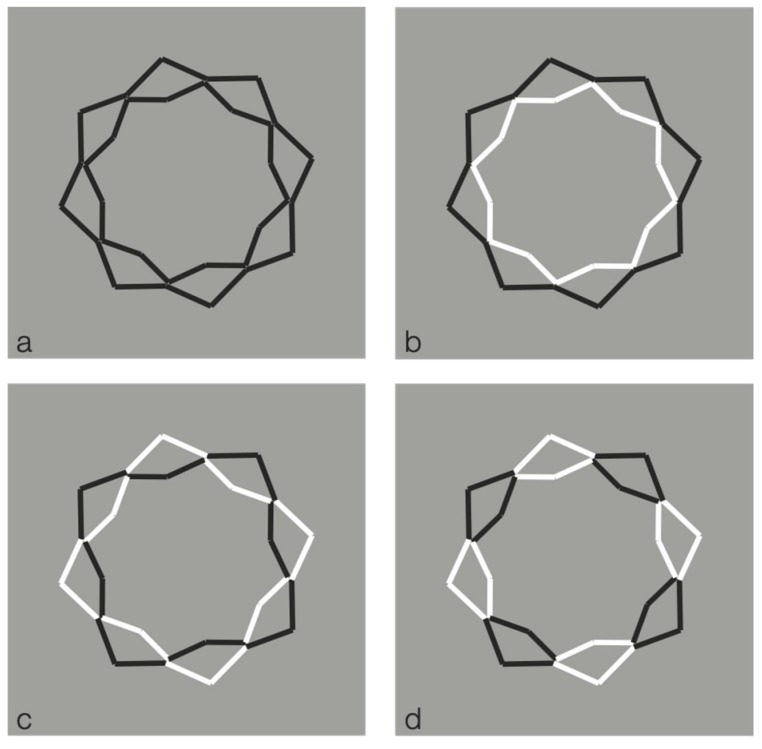
Different results on the basis of contrast polarity.

The strength of contrast polarity in terms of salience against other principles can be further perceived and appreciated in Figure 8 and Figure 9. 

Note that we are now introducing the term “salience,” as a phenomenal attribute imparting a strong highlighting effect. Under these conditions, contrast polarity is used as a special case of the similarity principle, i.e., one of the many possible kinds of similarity, but in the next figures, due to the salience effect, it can be considered as a special case of the accentuation principle.

It is worth emphasizing that among the similarities, the reversed contrast is, in our opinion, one of the strongest and likely the strongest factor, and, as it happens in Figure 9, this is due to its highlighting effect that immediately jumps to the eye. Here, starting from two nested eight-pointed stars, namely, from two simple, singular, symmetrical, and Prägnant figures, we can highlight and then pop out new configurations.

**Figure 10:**
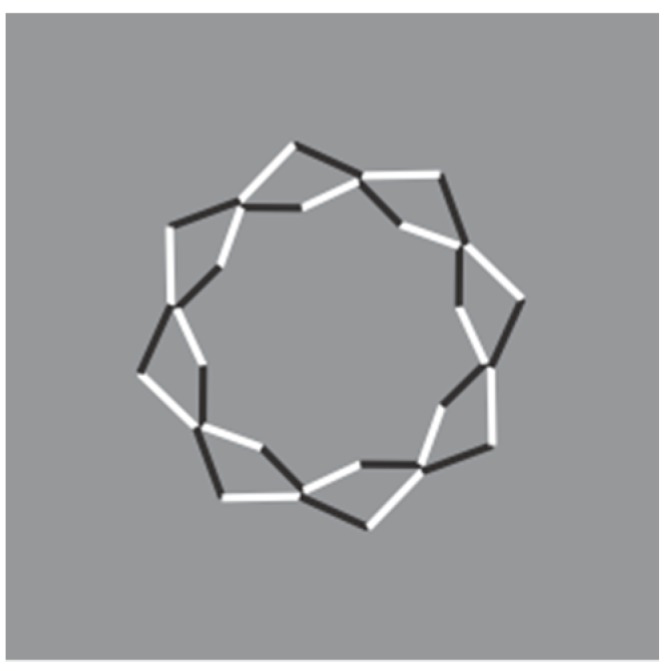
An intertwined flower-like spiral.

Figure 10 shows Figure 9a that becomes a sort of intertwined flower-like spiral without clean and univocal external boundaries.

**Figure 11:**
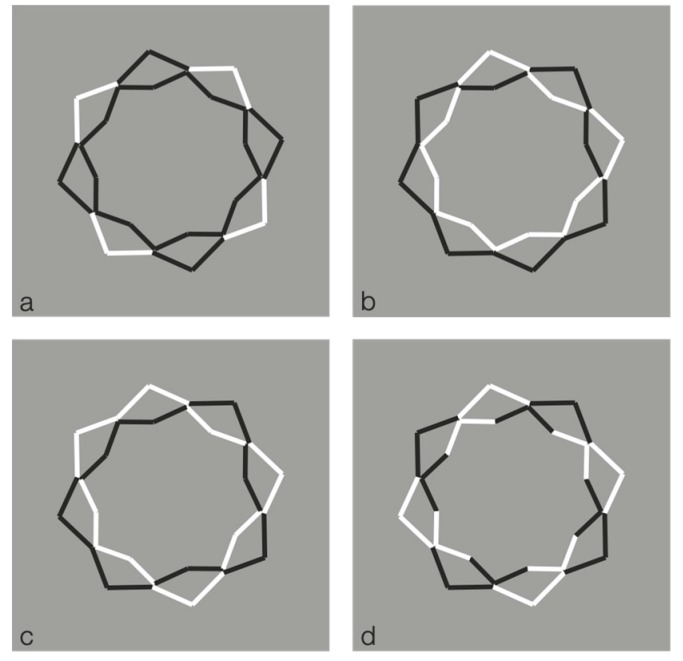
Iregular shapes from contrast polarity.

**Figure 12:**
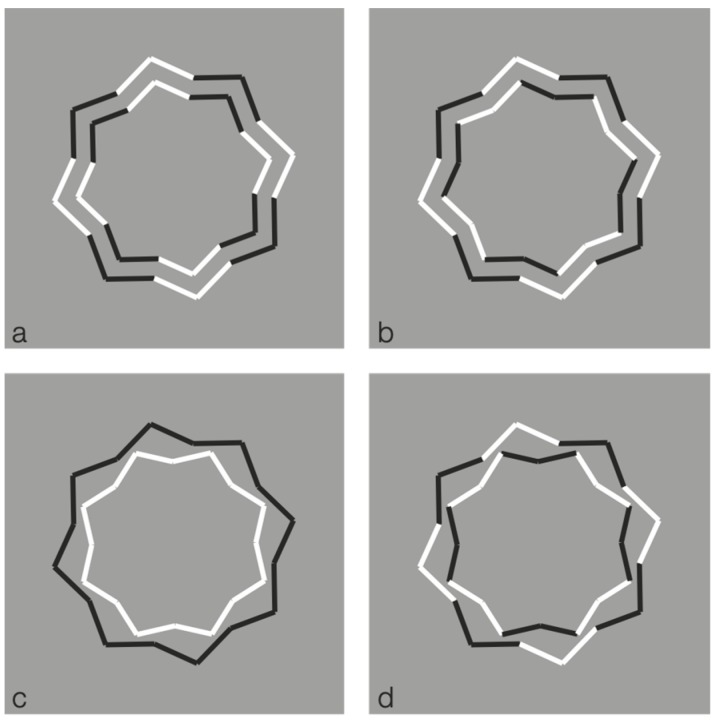
Regular and irregular shapes from reversed contrast.

Regularity and likelihood of the previous figures become irregular and unlikely in Figure 11 and, more efficiently, in Figure 12.

**Figure 13:**
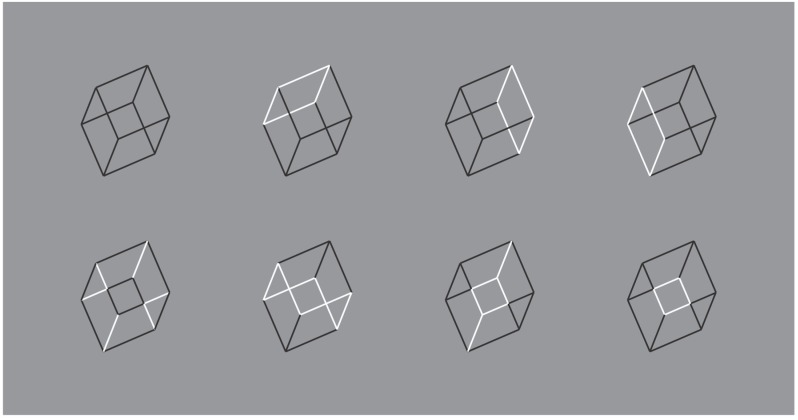
Different views of the Necker cube (first row) and weakened, disrupted, camouflaged, and invisible cube (second row).

From these examples, contrast polarity is not used as a similarity principle but more and more to accentuate and highlight new elements previously invisible and camouflaged. This idea, already discussed in our paper, will be developed in the next figures starting from the following variations of Necker cube (cf. Figure 13).

What is worthwhile to be considered in this figure is the set of cubes of the first row, where highlighting one view or the other, by means of reversed contrast, the same cube seems to be differently oriented in 3D space. The reversed contrast is now more related to accentuation principle than to similarity.

**Figure 14:**
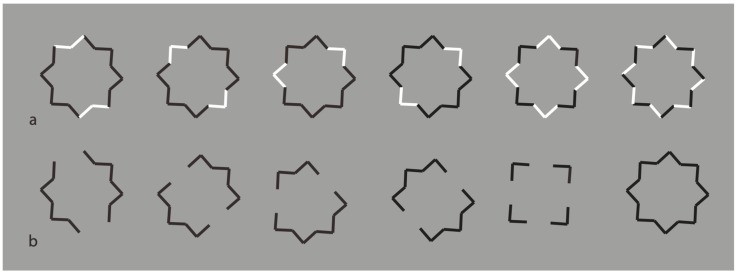
The black boundaries of a regular eight-pointed star discontinued by reversing the contrast polarity.

Given the salience effect, we finally explore limiting conditions, more directly related to simplicity and likelihood approaches. These conditions represent a further level of application of the reversed contrast, no more within multiple contours highlighting, disrupting, or camouflaging one or another possible configuration, but within a unique closed shape, more simple as possible, and where the possibility to perceive further inset subcomponent is very unlikely. These cases allow to test more appropriately simplicity and likelihood. Some of these conditions are illustrated in Figure 14.

Now, the black boundaries of a regular eight-pointed star have been discontinued by reversing the contrast polarity in different portions of each figure. In terms of grouping, the closure principle is mainly responsible for the perception of the eight-pointed star. Phenomenally, the uniqueness and wholeness of each star illustrated appear weakened or broken down. Each shape of the first row partially splits into two black and white adjacent components that are seen as belonging to two different objects. 

The most extensive black components reveal more easily the whole shape that, given the ungrouped white sides, do not appear as eight-pointed stars, but as the related shapes illustrated in Figure 14b, with inner gaps interrupting the boundary continuation. The removal of the white components improves the grouping of the black segments on the basis of the closure and good continuation principles. The missing sides do not affect the whole shape but appear as gaps favoring the good continuation of the modal sides and the amodal wholeness [12]. Given the black and white alternation of the sides, only the last condition of the first row appears as analogous to the star of the last condition of Figure 14b.

**Figure 15:**
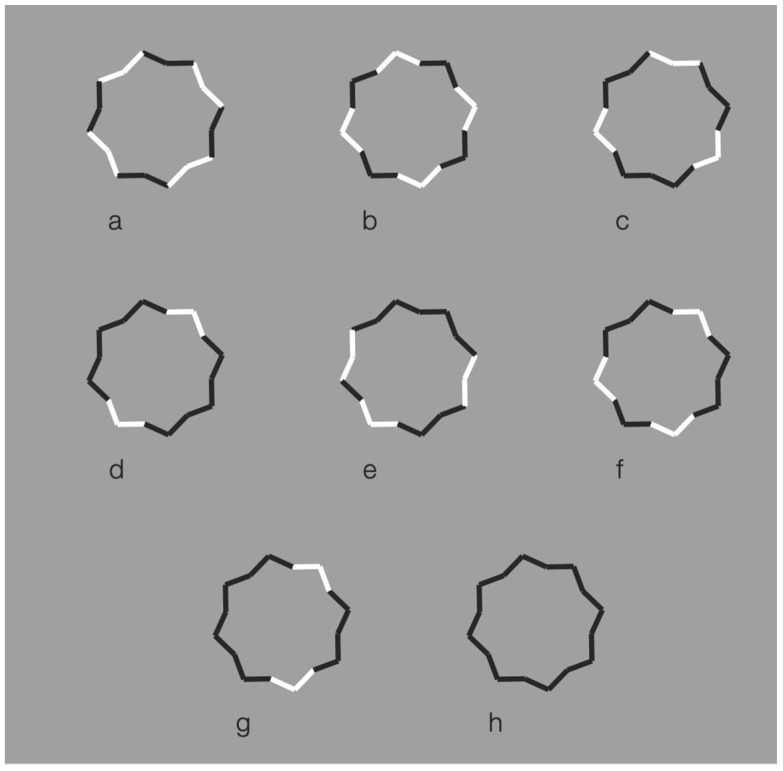
Further examples of the role played by contrast polarity in breaking the unitariness of a star.

Further examples are illustrated in Figure 15. Again, the reverse contrast breaks the oneness and the unitariness of the eight-pointed star and influences significantly its shape. New organizations emerge as follows: A concave polygonal shape rather than a star (Figure 15a); two rotated and perpendicular intersecting square shapes with illusory curved sides (Figure 15b); irregular shapes different from stars in Figure 15c–g. Figure 15h is the control.

Figure 15a,b display something that is worth noticing, namely the emergence of the concavity and of the convexity within the same geometrical star. As a matter of fact, the two figures are perceived as stars. However, the first appears as a sort of ‘eight-concave star’ or ‘eight-indented star.’ 

The breaking of the uniqueness and unitariness is very effective even though good continuation, closure, and Prägnanz together favor the grouping. The dissimilarity imparted by the reversed contrast split the figure. The question is: Why are these irregular and parceled solutions preferred to a simpler result like ‘a star with black and white edges?’ With this solution, both the whole shape and the local changes would have been preserved and saved. Even if this ideal solution is possible, it does not prevail.

These outcomes clearly challenge the theoretical assumptions of oneness, unitariness, symmetry, regularity, simplicity, minimization of description length, likelihood, and previous knowledge.

**Figure 16:**
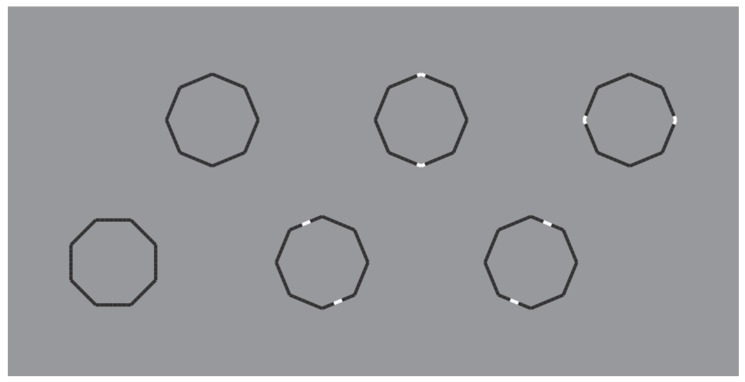
Octagons apparently different due to the accentuation imparted by contrast polarity.

These limiting conditions can be even more pushed over by drastically reducing the role of the discontinuities due to the reversed polarity and, therefore, by improving the good continuation and the similarity of remaining parts of the figure. This is the case of the polygons illustrated in Figure 16, where the perception of a polygon is the only possibility with the maximum likelihood.

In Figure 16 top-center, a regular octagon is perceived with the vertices oriented along the main directions of space (vertical and horizontal). By introducing achromatic discontinuities on two vertices placed along the vertical or on the horizontal axes of the polygon (second and third figure in the first row), the same kind of oriented regular octagon is seen. However, by introducing similar discontinuities in two opposite sides placed along an oblique axes (second and third figure in the second row), the polygons are now perceived as equal and, at the same time, different: Their shapes appear more similar to the first octagon of the second row, i.e., with the sides, rather than the vertices, oriented along the main directions of space. Moreover, the second and third octagons of the second row appear as oriented on opposite oblique directions with respect to the vertical axes.

**Figure 17:**
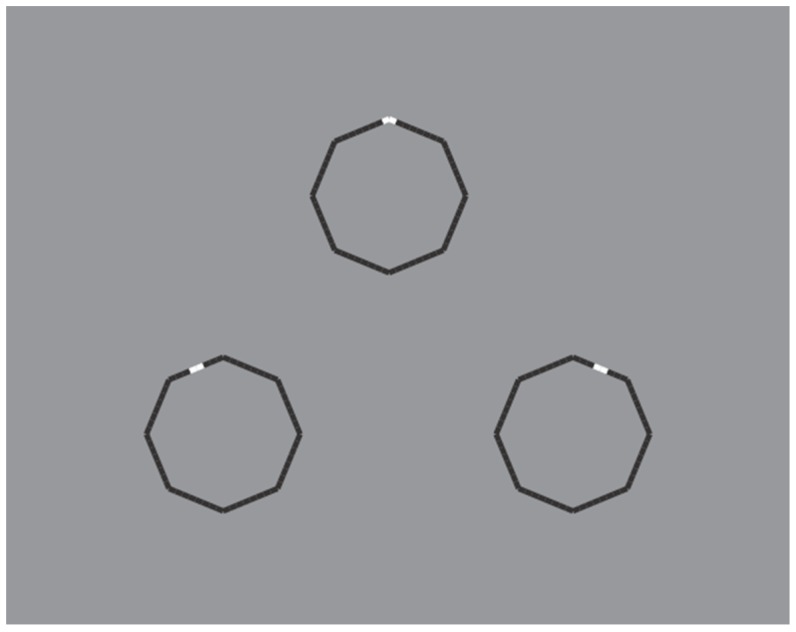
The octagons appear different due to a single white accentuation imparted by the contrast polarity.

Similar results can be produced with just a single white discontinuity (see Figure 17).

Unlike the stars of Figure 14 and Figure 15, the wholeness of the polygons is not broken down or disrupted. This effect mainly occurs due to the significant size reduction of the white components that let the good continuation and, hence, the unitariness and the wholeness of the boundaries at work. Phenomenally, the white discontinuities, placed on the vertices (angles) and sides, highlight and accentuate within the same shape different components (angles and sides) and object attributes.

The white dashes within the polygons behave phenomenally like accents inducing the pop out of one or another geometrical basic component of the polygon, i.e., angle or side. The white dashes do not represent the source of the disruption of the object, as shown in the previous figures, but behave like accents of a specific attribute of the figure that is ipso facto highlighted. This accent determines a change of the whole object, not only of its orientation or tilt, but of one of the two attributes. In other terms, the accent changes the nature and the geometry of the object. The accentuated pointedness and sidedness create two different polygons (same but different), one more pointed and the other flatter.

**Figure 18:**
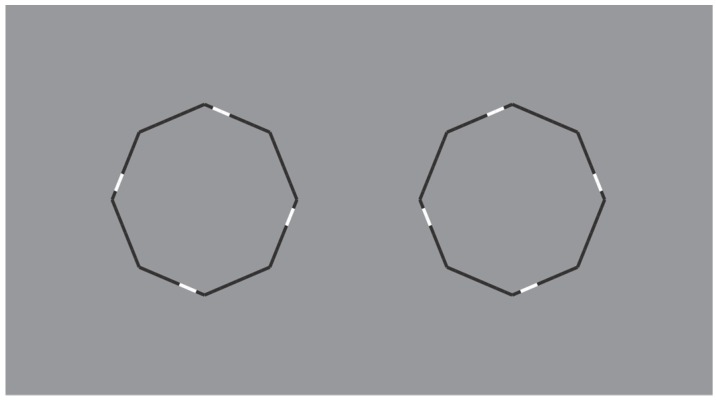
The octagons appear irregular and oriented in opposite directions.

The accentuation induced by contrast polarity can also disrupt the regularity of the octagons as illustrated in Figure 18. 

### Counter Comments: Reversed Contrast and Amodal Completion

Differently from van der Helm’s judgment, the stimuli presented in this section demonstrate the novelty of the way contrast polarity was used. 

It is surprising that van der Helm based his critiques only on the example produced by himself and not on our stimuli. This is unfair, and in our view, scientifically inappropriate, to quote the same words used by van der Helm. It would have been scientifically more correct to comment in detail on each of our figures.

**Figure 19:**
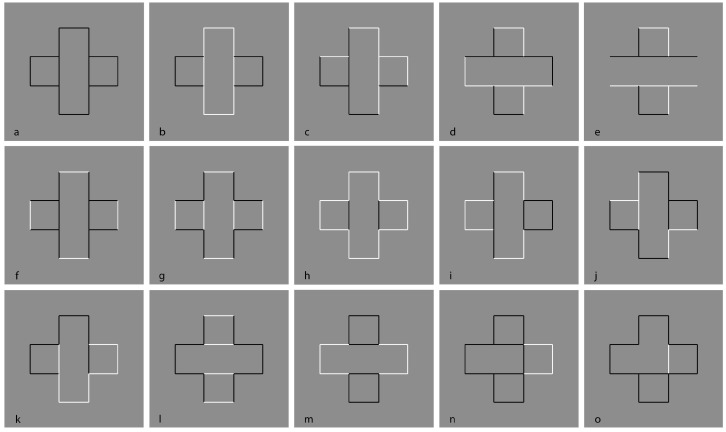
Conditions demonstrating the dominance of the contrast polarity over good continuation, T-junctions, regularity, simplicity, and likelihood.

**Figure 20:**
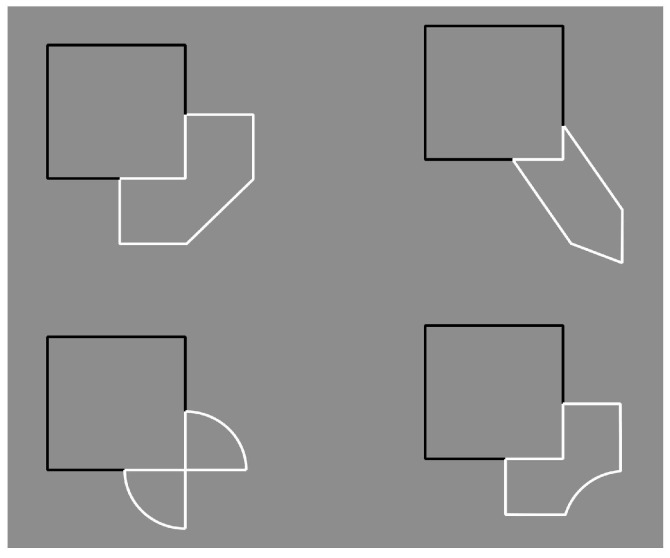
The contrast polarity reverses the amodal completion: The square is now perceived as partially occluded by the asymmetrical objects (for a control see Figure 21).

**Figure 21:**
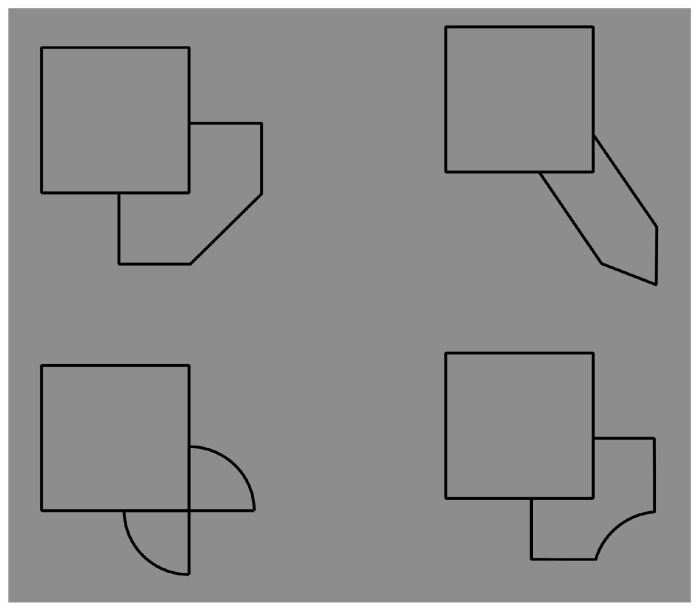
A control for Figure 20. The figures, partially occluded by the square, complete amodally as asymmetrical shapes.

Further interesting conditions for the discussion are shown in Figure 19, Figure 20 and Figure 21. In Figure 19, the reversed contrast is pitted in favor or against T-junctions, good continuation, closure, simplicity, and likelihood. A detailed description of this set of stimuli can be found in our paper. 

Unfortunately, van der Helm did not discuss these stimuli that represent the core of our work. Moreover, he did not notice the novelty of the I-junctions, more clearly shown in Figure 20 (Figure 21 is a control), where the continuation of contours is behind a contour with the same orientation. In other terms, two contours, although showing the same orientation, are perceived as two different contours where one appears to complete amodally behind the other. The effect of the I-junctions occurs against T-junctions, regularity, good continuation, closure, simplicity, likelihood. For a detailed description of this novel outcome see Pinna & Conti [2].

## 4. On Local vs. Global Assumption: Likelihood and Simplicity

Van der Helm judged and labelled three sentences of our paper as incorrect assumptions. The sentences are the following:

- The salience and visibility, derived by the largest amplitude of luminance dissimilarity imparted by contrast polarity, precedes any holist or likelihood organization due to simplicity/Prägnanz and Bayes’ inference.
- Contrast polarity was shown to operate locally, eliciting results that could be independent from any global scale and that could also be paradoxical. These results weaken and challenge theoretical approaches based on notions like oneness, unitariness, symmetry, regularity, simplicity, likelihood, priors, constraints, and past knowledge. Therefore, Helmholtz’s likelihood principle, simplicity/Prägnanz and Bayes’ inference were clearly questioned since they are supposed to operate especially at a global and holistic level of vision.
- The highlighting strength of contrast polarity determines even the grouping effectiveness against the global and holistic rules and factors expected by Helmholtz’s likelihood principle, simplicity/Prägnanz and Bayes’ inference.

A simple response to this judgement is that the three sentences are not assumptions but just evidence based on our results and on the phenomenology of the stimuli. On the contrary, we think that van der Helm’s judgement is based on an incorrect assumption. In fact, he did not comment on the three sentences on the phenomenology of our effects, i.e., by showing and demonstrating that our results are not consistent with our conclusion. Instead, he assumed as incorrect our sentence on the base of the following results obtained by van Lier:

*It is true that simplicity and likelihood approaches may aim to arrive at global stimulus interpretations, but a general objection against the above stance is that they (can) do so by including local factors as well. For instance, van Lier [16] presented a theoretically sound and empirically adequate simplicity model for the integration of global and local aspects in amodal completion.*

We think that this is unfair and scientifically inappropriate, in his own words. In fact, he continues:

A methodological objection is that Pinna and Conti introduced contrast polarity changes in stimuli but pitted these against alleged simplicity and likelihood predictions for the unchanged stimuli. As I specify next, this is unfair, and in my view, scientifically inappropriate.

Starting from our purpose—according to which contrast polarity has been used as the main grouping and ungrouping factor aimed to explore and test amodal completion as a visual phenomenon elicited by good continuation, T-junctions, closure, and regularity—this is not unfair and it is not scientifically inappropriate since our predictions are not based on unchanged stimuli but, on the contrary, on changed stimuli. For example, in the case of Figure 21 of our paper, we stated: “The breaking of the uniqueness and unitariness is very effective even though good continuation, closure, and, even, Prägnanz together favor the grouping. The dissimilarity imparted by the reversed contrast splits the figure. The question is: why are these irregular and parceled solutions preferred to a simpler result like ‘a star with black and white edges?’ With this solution, both the whole shape and the local changes would have been preserved and saved. Even if this ideal solution is possible, it does not prevail.”

A further van der Helm’s critique states as follows:

Pinna and Conti wrote “[If] we do not consider the contrast polarity as a constraint or as a prior, Bayes’ inference cannot easily explain these conditions” [2] (p. 12 of 32)—indeed, but why would we? Hence, they knowingly ignored the above flexibility and applied likelihood as if it is fundamentally blind to contrast polarity. Thereby, they missed the mark in their assessment of likelihood approaches.

We did not ignore the flexibility of the Bayesian approach; in fact we wrote: “Bayes’ inference cannot easily explain these conditions.” We just reported the most immediate prediction that could be made and leave open the point. Nevertheless, van der Helm based his judgement on vague conjecture and this cannot be accepted. On the contrary, it would have been acceptable whether he had explained our stimuli according to Bayesian inference. 

We do deny that this kind of explanation might be possible, we are not against Bayesian inference, but we just casted doubts about its effectiveness in the light of our stimuli.

In commenting on simplicity, van der Helm refers to Figure 2, introduced by himself, and draws conclusions on our work by stating: “Pinna and Conti knowingly ignored this and applied simplicity as if it is fundamentally blind to contrast polarity. Thereby, they missed the mark in their assessment of simplicity approaches”. We cannot accept this gratuitous judgment and critique based on general hypothesis, far away from our work. We really do not understand this attitude. We would have preferred a direct comment to each of our figures. This is much more useful to improve theories, useful for science.

Anyway, we have never said that simplicity is blind to contrast polarity. We do not believe so. Our phenomenological background is based on the notion of simplicity. Our point was just focused on the conditions we studied. As a matter of fact, our results put to the test these important theories and approaches in line with Popper’s falsificationism. This is scientifically appropriate.

## 5. On the Equivalence between Simplicity and Likelihood

Van der Helm quoted the unique sentence in our paper where he thinks we claim the equivalence between simplicity and likelihood: “[...] the visual object that minimizes the description length is the same one that maximizes the likelihood. In other terms, the most likely hypothesis about the perceptual organization is also the outcome with the shortest description of the stimulus pattern.” [2] (p. 3 of 32). He also wrote: “This is an extraordinary claim”.

As a first reply to this comment, we can confidently say that it was far from our purpose to demonstrate or discuss the supposed equivalence. Our purposes were very different as described in detail in the first section of the present work. Second, the supposed equivalence is just related and based on our conditions only without any further generalization. In our opinion, based on our results, simplicity and likelihood do not deliver different predictions. For van der Helm, it would have been easier to indicate possible differences to give a more effective contribution.

We cannot accept that van der Helm can attribute to us generalizations never mentioned. Again, the supposed equivalence is implicit and restricted to only our conditions. We did nothing to prove or disprove this equivalence being far from our purposes. Third, in any case, as acknowledged even by van der Helm, this equivalence is not an extraordinary claim. Other scientists (see below) are in favor of some sort of equivalence.

According to Helmholtz’s likelihood principle, the sensory input is organized into the most probable distal object or event consistent with the sensory data (the proximal stimulus). This principle chooses the most likely true interpretation and assumes that the visual system is highly veridical in terms of the external world. From an evolutionary point of view, if the visual system were not veridical, it would probably not have survived during the evolution. 

On the other hand, the simplicity principle does not experience these problems, because it does not aim specifically at veridicality. The simplicity and the likelihood principle are two competing theories [5,17,18,19] of perceptual organization and visual coding, which are difficult to settle because neither of the key elements were clearly defined. The general difference between the two is related to the fact that the visual system, in the case of the simplicity, obeys a more general principle of economy, while in the case of the likelihood, it obeys a general principle of probability. 

Nevertheless, these two terms might be only apparently different or may be considered as two sides or two different ways of considering the same visual process. Mach [20] suggested that vision acts in conformity with the principle of economy, and, at the same time, in conformity with the principle of probability. Chater [21] demonstrated mathematically that these key elements can be unified and considered equivalent within the theory of Kolmogorov complexity [22,23,24,25,26,27]. 

Feldman [28,29,30,31] presented a simplicity approach, called minimal model theory, and, in agreement with Chater [21], suggested that the visual interpretation, whose description is of minimum length, is the one that most likely is also the most veridical. Usually, the tendency of choosing a visual object that minimizes the description length is the same as the tendency of choosing a hypothesis that maximizes the likelihood. In brief, the most likely hypothesis about perceptual organization is, at the same time, the objects supporting the shortest description of the stimulus. 

Far from this controversy, the point is here that we firmly reject van der Helm’s attribution of a supposed general equivalence between the two principles.

## 6. Conclusions

We discussed and counter-commented van der Helm’s [1] comments on Pinna and Conti [2] going into detail of his critiques and rejecting all of them point-by-point. We proceeded by summarizing hypotheses and discussion of the previous work, then commenting on each critique through old and new phenomena and clarifying the meaning of our previous conclusions. It is a pity that van der Helm’s comments were not directly related to our stimuli. This would have stimulated a more effective discussion and possibly some new ideas. Anyway, we hope that this exchange of views can stimulate other scientists to further develop theories and phenomena related to amodal completion and reversed contrast. We are convinced about the importance of these intriguing objects, not fully explained yet, and that deserve to be further studied through different perspectives. Finally, we hope that Bayesian simulations can cast new light on the hypothesis presented in our paper and in this discussion.
